# Chemotropic signaling by BMP7 requires selective interaction at a key residue in ActRIIA

**DOI:** 10.1242/bio.042283

**Published:** 2019-06-17

**Authors:** Jeanette C. Perron, Alcina A. Rodrigues, Nirupama Surubholta, Jane Dodd

**Affiliations:** 1 Department of Pharmaceutical Sciences, St. John's University, Queens, NY 11439, USA; 2 Departments of Physiology & Cellular Biophysics and Neuroscience, Columbia University, New York, NY 10032, USA

**Keywords:** BMP7, ActRIIA, Growth cone collapse, Chemotaxis, PI3K

## Abstract

BMP7 evokes acute chemotropic PI3K-dependent responses, such as growth cone collapse and monocyte chemotaxis, as well as classical Smad-dependent gene transcription. That these divergent responses can be activated in the same cell raises the question of how the BMP-dependent signaling apparatus is manipulated to produce chemotropic and transcriptional signals. RNA interference and site-directed mutagenesis were used to explore functional and structural BMP receptor requirements for BMP7-evoked chemotropic activity. We show that specific type II BMP receptor subunits, ActRIIA and BMPR2, are required for BMP7-induced growth cone collapse in developing spinal neurons and for chemotaxis of monocytes. Reintroduction of wild-type ActRIIA into monocytic cells lacking endogenous ActRIIA restores BMP7-evoked chemotaxis, whereas expression of an ActRIIA K76A receptor variant fails to rescue. BMP7-evoked Smad-dependent signaling is unaffected by either ActRIIA knockdown or expression of the ActRIIA K76A variant. In contrast, BMP7-evoked PI3K-dependent signaling is significantly disturbed in the presence of ActRIIA K76A. These results support a model for selective engagement of chemotropic BMPs with type II BMP receptors, through specific residues, that results in strict regulation of PI3K-dependent signal transduction.

This article has an associated First Person interview with the first author of the paper.

## Abstract (precis)

**Summary:** Chemotropic BMPs, typified by BMP7, mediate selective receptor recruitment and transduction of PI3K-dependent intracellular signals through interaction with a key residue in the ActRIIA type II BMP receptor.

## INTRODUCTION

Bone Morphogenetic Proteins (BMPs) are a large protein family, belonging to the TGFβ superfamily of secreted factors (Miyazono et al., 2010; Heldin and Moustakas, 2016). Although first identified for the ability to induce bone formation (Urist and Strates, 1971), BMPs have since been found to play important and diverse roles from embryogenesis to adulthood. Thus, BMP signaling is critical for many developmental processes, such as gastrulation, patterning of the developing spinal cord, axon guidance and organogenesis (Hegarty et al., 2013; Meyers and Kessler, 2017). In adult tissues BMPs regulate proliferation, apoptosis, bone homeostasis and immune responses (Heldin and Moustakas, 2016; Katagiri and Watabe, 2016). Moreover, dysregulation of BMP signaling is associated with various pathologies involving neurological, cardiovascular and pulmonary systems (Wang et al., 2014; Brazil et al., 2015; Gallo MacFarlane et al., 2016). In light of this diversity, much progress has been made elucidating BMP ligand/receptor interactions and revealing components of signaling cascades downstream of these interactions (Derynck and Zhang, 2003; Miyazono et al., 2010; Heldin and Moustakas, 2016).

The many diverse actions of the >20 known BMPs are mediated by a relatively small number of type I and type II BMP receptor serine/threonine kinase subunits, subsets of which are arranged in distinct tetrameric complexes (Nickel et al., 2009; Yadin et al., 2016). Four type I receptors [ALK1, ALK2, ALK3 (BMPRIA) and ALK6 (BMPRIB)] and three type II receptors (ActRIIA, ActRIIB and BMPR2) are utilized by BMPs. In addition, the function of numerous cell surface co-receptors as well as crosstalk with other signaling pathways has been demonstrated for a number of TGFβ superfamily members (Zhang, 2017; Nickel et al., 2018).

The downstream signaling mediators classically associated with BMP receptor activation are the type I BMP receptor-regulated Smads (Smad1/5/8), activation of which leads to transcriptional regulation of BMP target genes (Feng and Derynck, 2005; Miyazono et al., 2010; Nishimura et al., 2012). In addition to the canonical Smad-dependent pathway, a number of non-canonical or alternative signaling pathways linked to type I BMP receptors lead to transcriptional responses, including p38 and ERK MAPK pathways (Heldin and Moustakas, 2016; Zhang, 2017). More recently, non-Smad, non-transcriptional, BMP-dependent signaling mediators have been reported, including PI3K and LIMK, that link BMP signals to the regulation of cytoskeletal organization (Foletta et al., 2003; Wen et al., 2007; Perron and Dodd, 2011). Thus, in contrast to transcriptional pathways that require type I BMP receptors; regulators of cytoskeletal organization appear to be linked to activation of type II BMP receptors.

Despite the progress outlined above, the role of distinct receptor subtypes in different BMP functions and the requirements for assembly of specific BMP receptor subunits into complexes that lead to downstream signaling and cellular responses remain unclear. The chemotropic functions of BMP7 provide a model for investigating these issues. At sufficient concentrations, all BMPs stimulate Smad1/5/8 phosphorylation (pSmad1/5/8), which over a relatively slow time course regulates gene transcription. In contrast, a small subset of BMPs (BMP2, BMP4, BMP7 and BMP9; ‘chemotropic BMPs’) stimulates non-Smad pathways, leading to rapid onset of chemotropic activities such as axon guidance and chemotaxis (Augsburger et al., 1999; Wen et al., 2007; Gamell et al., 2008; Perron and Dodd, 2009, 2011, 2012). Moreover, at low concentrations only the non-canonical pathway is activated. Thus, in addition to Smad1/5/8 stimulation, chemotropic BMPs stimulate PI3K-dependent Akt phosphorylation (pAkt). Non-chemotropic BMPs, such as BMP6, do not exhibit acute chemotropic activity at any concentration and have no effect on pAkt levels. BMP7 stimulates growth cone collapse, monocyte chemotaxis and a PI3K-dependent increase in pAkt levels at concentrations at which phosphorylation of Smad1/5/8 is undetectable (Perron and Dodd, 2009, 2011). How chemotropic BMPs interact with receptor complexes selectively to activate type II BMP receptor-dependent signaling at low concentrations and to activate Smad-dependent signaling only at higher concentrations remain open questions.

The four identified chemotropic BMPs belong to three different structural subgroups within the BMP family and each subgroup displays different affinity profiles for both type I and type II BMP receptors suggesting that binding affinity alone does not account for the shared activity of chemotropic BMPs (Kirsch et al., 2000; Allendorph et al., 2007; Yadin et al., 2016). Our previous work has demonstrated that BMP-evoked chemotropic activity is independent of type I BMP receptor activity and Smad1/5/8 activation, highlighting the importance of type II BMP receptors in mediating the chemotropic functions of BMPs (Perron and Dodd, 2009, 2011). Indeed, loss of ActRIIA or BMPR2, but not ActRIIB, expression inhibits BMP7-evoked chemotaxis, revealing a requirement for specific type II BMP receptor subunits for transduction of BMP7-evoked chemotaxis (Perron and Dodd, 2009). These findings support a model in which chemotropic BMPs recruit or assemble specific type II BMP receptor subunits that selectively mediate chemotropic functions. Further support for this model comes from single residue-swapping experiments in which BMP6, a BMP with no measureable chemotropic activity, was converted into a chemotropic BMP by exchanging *Gln^48^* in BMP6 with the BMP7-equivalent amino acid, *Arg^48^* (Perron and Dodd, 2012). A structural model of BMP7 bound to the extracellular domain (ECD) of ActRIIA [Protein Data Bank (PDB) ID: 1LX5; Greenwald et al., 2003], reveals that a likely site of interaction with BMP7 and BMP6 Q48R but not wild-type BMP6 is an amino acid residue in ActRIIA, *Lys^76^* (Perron and Dodd, 2012). This site is located within an identified ‘hot spot’ of affinity and specificity for type II BMP receptor binding (Allendorph et al., 2007; Song et al., 2010; Yadin et al., 2016).

In the present study, we have used RNA interference (RNAi) with short-hairpin RNAs (shRNAs) targeting type II BMP receptors and performed rescue experiments with shRNA-resistant cDNA expression in growth cone collapse and monocyte chemotaxis assays to explore type II BMP receptor requirements specific for BMP7-evoked chemotropic activities. We have previously shown that all three type II BMP receptors are expressed in both WEHI cells and dorsal spinal cord (DSC) neurons (Perron and Dodd, 2009, 2011). We now show that both ActRIIA and BMPR2, but not ActRIIB, are required for BMP7-mediated growth cone collapse of DSC neurons. BMP7-evoked monocytic chemotaxis that is inhibited by selective loss of ActRIIA expression is rescued by introduction of an shRNA-resistant ActRIIA cDNA, whereas expression of an ActRIIA K76 variant was unable to rescue BMP7-evoked monocyte chemotaxis. Moreover, overexpression of ActRIIA K76 variant subunits interfered with BMP7-stimulated chemotaxis even in the presence of endogenous ActRIIA. Substitution of *Lys^76^* in ActRIIA with *Ala^76^* or *Glu^76^* also eliminated BMP7-stimulated increases in pAkt. In contrast, concurrent BMP7-induced increases in pSmad1/5/8 levels were not affected by the loss of ActRIIA or expression of the ActRIIA K76 variants. Our results suggest a model in which ActRIIA and BMPR2 subunits are selectively recruited by the chemotropic BMP, BMP7, to mediate growth cone collapse and monocyte chemotaxis and that specific engagement with *Lys^76^* in ActRIIA by BMP7 is linked to selective regulation of PI3K-dependent signaling.

## RESULTS

### Type II BMP receptor shRNA vectors depress expression in DSC neurons

To determine whether the BMP receptor subunit selectivity observed in monocyte chemotaxis is a characteristic shared by other BMP-evoked chemotropic activities, such as axon orientation and growth cone collapse, we used GFP-expressing shRNA vectors, previously validated in monocytes (Perron and Dodd, 2009), that target individual type II BMP receptors in cultures of DSC neurons. These cultures, like most primary neurons, are notoriously difficult to transfect using standard lipofection techniques.

**Fig. 1.**
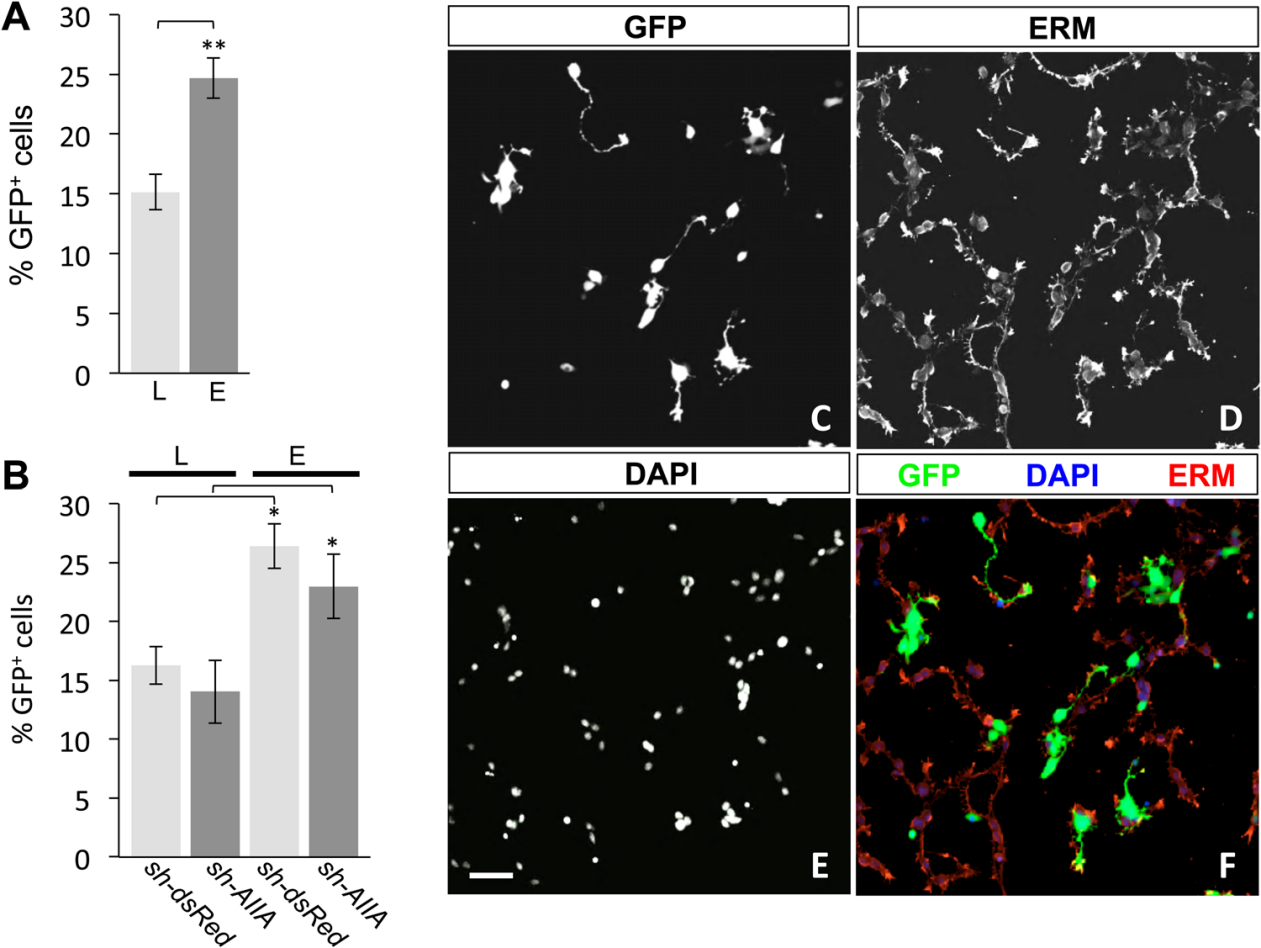
**Transfection of shRNA vectors in DSC neurons by lipofection and electroporation.** (A) Combined transfection efficiency assessed by the percentage of GFP**^+^** neurons (mean±s.e.m.) in dsRed^Δ^DSC and AIIA^Δ^DSC cultures by lipofection (L, 15.2%±1.5%) or electroporation (E, 24.7%±1.7%). Electroporation significantly improved transfection efficiency compared with lipofection (**P<0.002, *n*=3) (Student's two-tailed *t*-test). (B) Transfection efficiencies (% GFP**^+^** neurons; mean±s.e.m.; *n*=3 for each condition) for dsRed^Δ^DSC and AIIA^Δ^DSC cultures show no vector-specific differences in neurons transfected by either lipofection (dsRed^Δ^DSC, 16.3%±1.6%; AIIA^Δ^DSC, 14.0%±2.7%) or electroporation (dsRed^Δ^DSC, 26.4%±1.9%; AIIA^Δ^DSC, 23.0%±2.7%). In contrast, transfection efficiencies revealed that in dsRed^Δ^DSC and AIIA^Δ^DSC cultures, the percentage of GFP^+^ neurons increased by 62% and 63%, respectively, in electroporated cultures compared with transfection by lipofection (*P<0.05, *n*=3 for both conditions) (Student's two-tailed *t*-test). (C–F) Dissociated dsRed^Δ^DSC culture labeled for (C) GFP, (D) ERM and (E) DAPI. Merged image (F) shows GFP expression in electroporated neurons (green), ERM labeling localized to the cellular membranes (red) and DAPI staining the nuclei (blue). Scale bar: 50 μm.

In DSC cultures transfected with either *sh-dsRed* (dsRed^Δ^DSC), a functional negative control shRNA plasmid targeting red fluorescent protein, or *sh-AIIA* (AIIA^Δ^DSC), targeting ActRIIA, lipofection methods produced transfection efficiencies at an average of 15% (Fig. 1A). There was no difference in the percentage of GFP^+^ cells between dsRed^Δ^DSC and AIIA^Δ^DSC cultures transfected by lipofection, exhibiting transfection efficiencies of 16% and 14%, respectively (Fig. 1B). To improve transfection efficiency in DSC cultures, we explored the feasibility of whole-embryo electroporation for expression of shRNA vectors in dorsal spinal cord tissue. Briefly, DNA was injected into the central canal of E13 rat spinal cords and the dorsal spinal tissue was then electroporated, isolated and plated in dissociated culture (Fig. 1C–F). Electroporation of *sh-dsRed* or *sh-AIIA* resulted in large numbers of GFP^+^ cells with combined transfection efficiencies averaging 25% (Fig. 1A). Transfection efficiencies following electroporation were similar for dsRed^Δ^DSC and AIIA^Δ^DSC cultures (26% and 23% GFP^+^, respectively; Fig. 1B). Comparison of shRNA vector expression by lipofection and electroporation demonstrated that electroporation methods generate significantly higher transfection efficiencies in dissociated DSC cultures than those achieved by lipofection techniques (Fig. 1A; L versus E, 63% increase, **P<0.002, Student's two-tailed *t*-test).

**Fig. 2.**
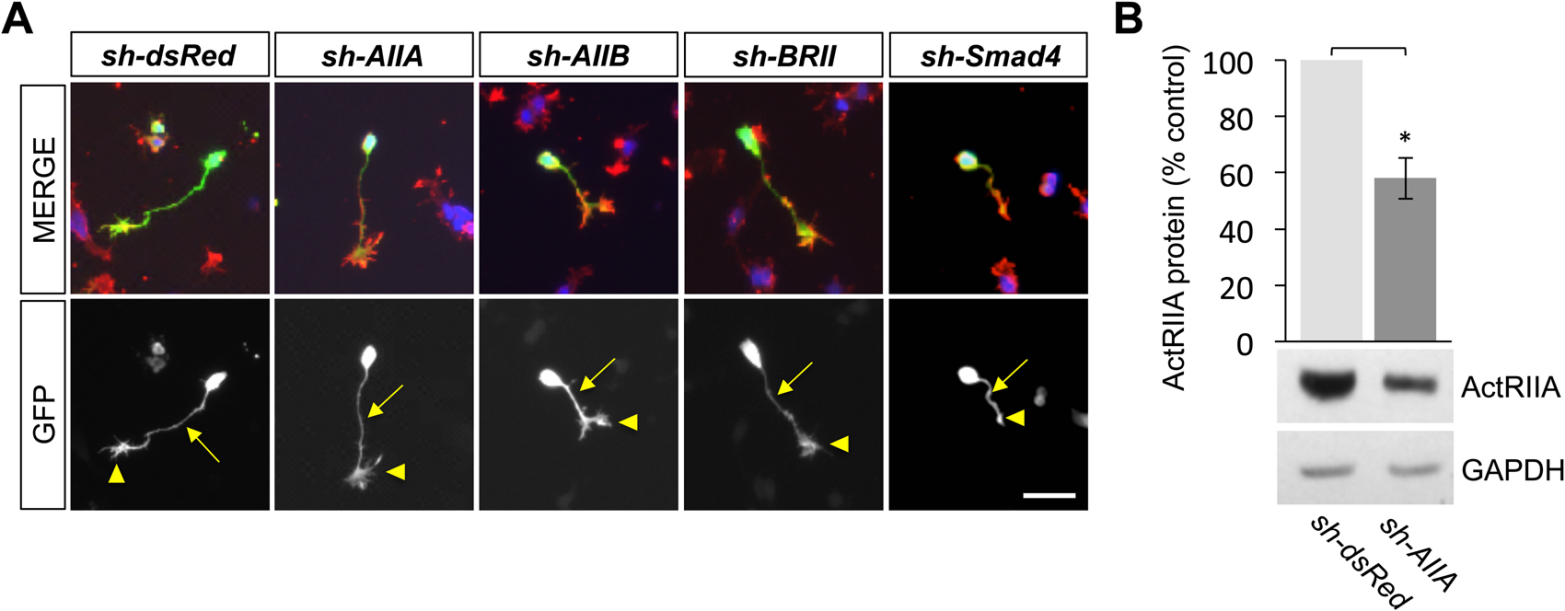
**Growth cone expression of type II BMP receptor-targeted shRNA vectors in DSC neurons.** (A) Representative images of DSC neurons electroporated with the indicated shRNA vectors in dissociated culture labeled for GFP (green), ERM (red) and DAPI (blue). The presence of GFP reflects expression of each of the shRNA vectors in cultured DSC neurons including in the axonal processes (arrows) and growth cones (arrowheads). In the far right column, a Smad4^Δ^DSC neuron is shown, treated with 50 ng/ml BMP7 for 30 min, and represents a collapsed growth cone (arrowhead). Scale bar: 50 μm. (B) Western blots of dsRed^Δ^DSC or AIIA^Δ^DSC lysates probed with an anti-ActRIIA antibody. Detection of GAPDH provided a loading control. Electroporation of *sh-AIIA* in DSC cultures decreases ActRIIA protein expression to 58%±7.4% of the expression levels in GFP-enriched, dsRed^Δ^DSC lysates (mean±s.e.m.; *n*=4; *P<0.05) (Student's two-tailed *t-*test).

We previously showed that the type II BMP receptor-targeting shRNA vectors efficiently and selectively inhibit BMP receptor expression in monocytes (Perron and Dodd, 2009). GFP expression was observed in DSC neurons electroporated with each of the shRNA knockdown vectors used in this study and was detected not only in the cell bodies, but also in all neuronal processes, including, importantly, the tips of growth cones (Fig. 2A). Western blot analysis demonstrates that *sh-dsRed* has no effect on the expression of ActRIIA compared to that in untransfected DSC neurons (Fig. S1). To ensure that the shRNA vectors similarly inhibit type II BMP receptor expression in embryonic DSC neurons, cultures of dsRed^Δ^DSC and AIIA^Δ^DSC were sorted by FACS to enrich for GFP^+^ neurons and western blot analysis was performed on whole-cell lysates. The lysates are enriched for GFP^+^ DSC neurons and, thus, underestimate the extent of ActRIIA knockdown in individual neurons. Nevertheless, western blots probed with an anti-ActRIIA antibody showed a 42% reduction in ActRIIA expression in AIIA^Δ^DSC lysates compared with dsRed^Δ^DSC lysates (Fig. 2B). The same lysates probed with anti-ActRIIA, anti-ActRIIB and anti-BMPR2 antibodies showed that the levels of the remaining type II BMP receptor subtypes were not affected by knockdown of ActRIIA (Fig. S2).

### Selective utilization of type II BMP receptors underlies BMP7-evoked growth cone collapse

**Fig. 3.**
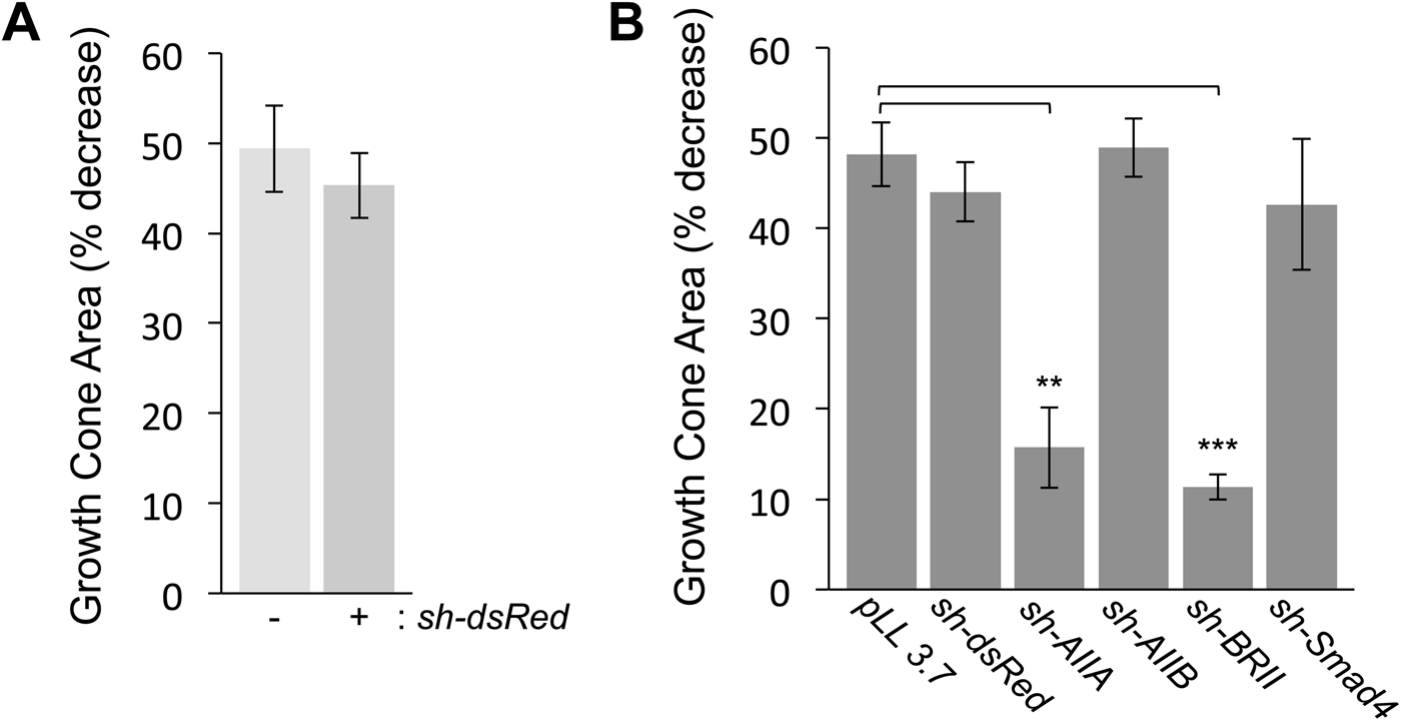
**BMP7-evoked growth cone collapse in DSC neurons in the presence of control and type II BMP receptor shRNA vectors.** (A) The percentage of growth cone area decrease [GCAD=(100–{[(GC area in the presence of BMP7–control GC area)/control GC area]×100}); mean±s.e.m.] in dsRed^Δ^DSC neurons stimulated by 50 ng/ml BMP7 for 30 min is not affected by expression of the non-specific shRNA vector, *sh-dsRed* (GCAD=48.3%±3.3%) when compared with untransfected, GFP^−^ neurons (GCAD=49.4%±4.8%) in the same culture (*n*=3). (B) Growth cone area decrease (mean±s.e.m.) in response to 50 ng/ml BMP7 was measured for shRNA-transfected GFP^+^ neurons only. Expression of pLL3.7, the shRNA parent vector, (GCAD=48.2%±3.5%; *n*=3) or knockdown of ActRIIB (AIIB^Δ^DSC, GCAD=54.4%±3.3%; *n*=3) or Smad4 (Smad4^Δ^DSC, GCAD=42.6%±7.2%; *n*=3) did not interfere with BMP7-evoked growth cone collapse. In contrast, BMP7-evoked growth cone collapse was significantly inhibited in AIIA^Δ^DSC (GCAD=15.7%±4.4%; *n*=4, **P<0.005) and BRII^Δ^DSC cultures (GCAD=11.3%±1.4%; *n*=3, ***P<0.001) (Student's two-tailed *t-*test).

BMP7 elicits growth cone collapse in DSC cultures (Augsburger et al., 1999; Perron and Dodd, 2011). To determine first whether electroporation of shRNA constructs alone interferes with BMP7-evoked growth cone collapse, dsRed^Δ^DSC neurons were incubated in the presence or absence of BMP7 and the percentage of growth cone area decrease (GCAD) was assessed. In dsRed^Δ^DSC (GFP^+^) neurons, GCAD was 49%, compared with 45% in untransfected (GFP^−^) neurons in the same culture (Fig. 3A). Thus, expression of the control *sh-dsRed* vector does not affect the ability of BMP7 to stimulate growth cone collapse.

To determine whether growth cone collapse evoked by BMP7 requires activity of specific type II BMP receptor subunits, receptor shRNA vectors targeting ActRIIA, ActRIIB and BMPR2, as well as dsRed and empty vector (pLL 3.7) negative controls, were expressed individually in DSC neurons by electroporation and incubated in the presence and absence of BMP7. Importantly, GFP is detected only in transfected neurons (Fig. 1C,F and Fig. S1) and only growth cones expressing GFP^+^ were analyzed (Figs 2A and 3B). BMP7-evoked decreases in growth cone area relative to untreated controls were observed in DSC neurons transfected with pLL 3.7 (GCAD=48%), *sh-dsRed* (GCAD=44%) and *sh-AIIB* (GCAD=49%) (Fig. 3B). In contrast, expression of *sh-AIIA* and *sh-BRII* resulted in only a 15% and 11% decrease in growth cone area, respectively (Fig. 3B), indicating that growth cone collapse evoked by BMP7 requires ActRIIA and BMPR2 but not ActRIIB subunits.

To assess the contribution of type I BMP receptor Smad-dependent signaling in BMP7-evoked growth cone collapse, Smad4, a co-factor required for Smad-dependent transcriptional responses, was inhibited by RNAi. In Smad4^Δ^DSC cultures, BMP7-evoked growth cone collapse was not affected (Fig. 3B). ActRIIA and BMPR2 are also selectively required for BMP7-stimulated monocyte chemotaxis (Perron and Dodd, 2009), suggesting that BMP7-evoked chemotropic activities across distinct cell types are mediated by a conserved arrangement of type II BMP receptor subunits and do not require type I BMP receptor-dependent Smad signaling.

### A single amino acid substitution in ActRIIA selectively disturbs BMP-evoked chemotropic activity

**Fig. 4.**
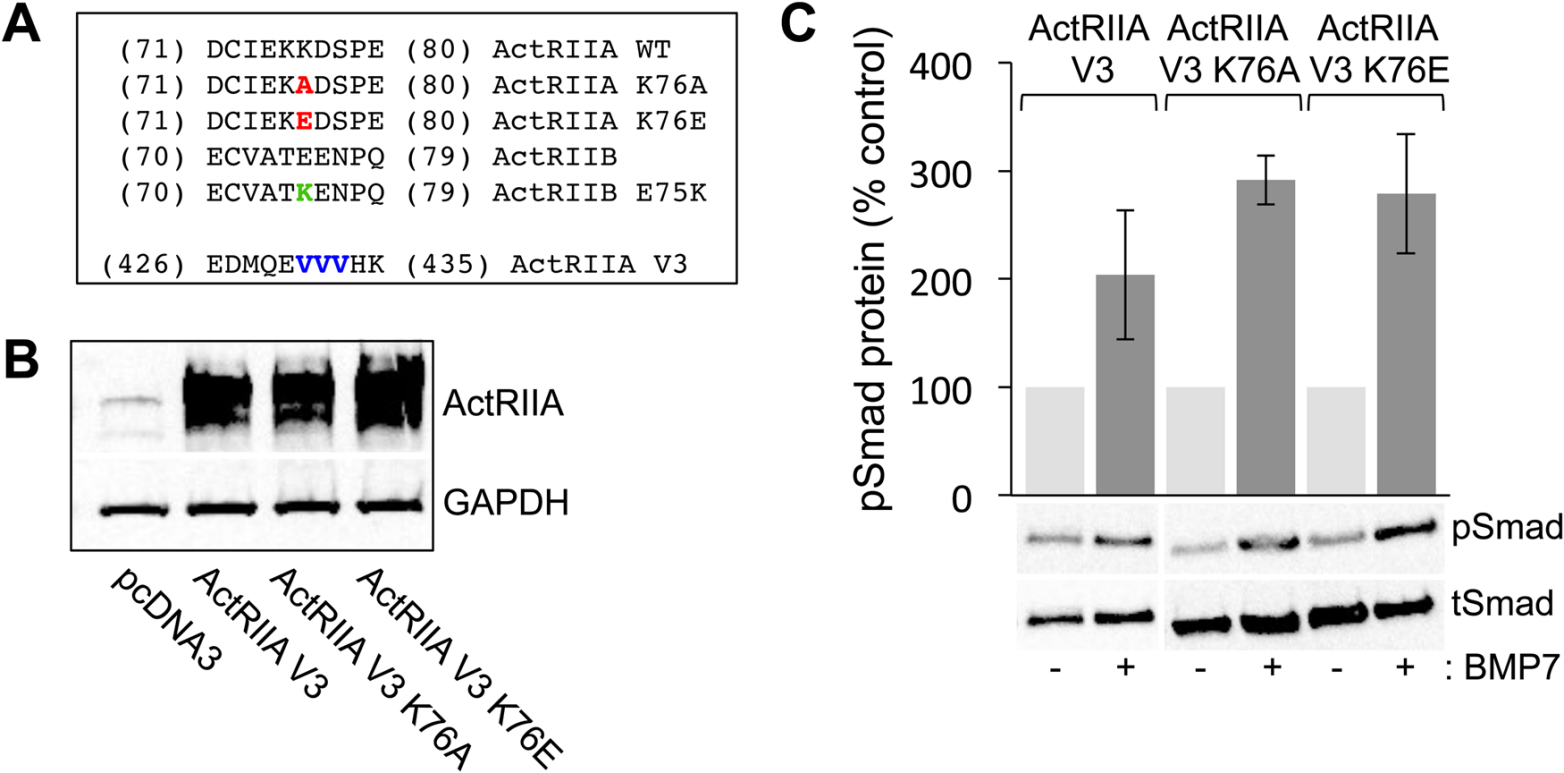
**Site-directed mutagenesis of K76 in ActRIIA does not impede variant receptor expression or BMP7-stimulated Smad1/5/8 phosphorylation.** (A) Amino acid sequences of mouse ActRIIA in the region of the K76 (red) and V3 (blue) mutations and mouse ActRIIB in the region of the E75 (green) mutation. Numbering corresponds to the amino acid number: for the K76 and E75 mutations according to PDB ID: 1LX5 and for the V3 mutations according to NCBI accession no.: NP_031422.3 (Fig. S3). The mutations at position 76 in ActRIIA replace lysine (K) with either alanine (A) or glutamic acid (E). The mutation at position 75 in ActRIIB replaces glutamic acid with lysine. The mutations at positions 431 to 433 do not replace the three valine (V) residues in the amino acid sequence but rather alter the nucleotide sequence to create an ActRIIA cDNA that is resistant to *sh-AIIA* while maintaining the amino acid sequence. (B) Western blots of whole-cell C2C12 lysates, transfected with pcDNA3, ActRIIA V3, ActRIIA V3 K76A or ActRIIA V3 K76E, were probed with an anti-ActRIIA antibody. Detection of GAPDH provided a loading control. (C) Quantification of western blots of transfected whole-cell C2C12 lysates incubated with or without 50 ng/ml BMP7 probed with a phospho-specific Smad1/5/8 antibody. Detection of total Smad1 provided a loading control. Densitometric analysis (mean±s.e.m.; *n*=2) shows an increase in response to BMP7 in cells expressing ActRIIA V3 (104% over control), ActRIIA V3 K76A (191% over control) and ActRIIA V3 K76E (179% over control).

The conversion of BMP6 into a chemotropic BMP by changing a single amino acid located within the ‘knuckle’ epitope of the BMP/type II BMP receptor-binding interface (Perron and Dodd, 2012) led us to consider the importance of reciprocal residues and structural requirements for chemotropic activity in individual BMP receptor subunits. Given the wealth of information available for BMP7 and the ActRIIA extracellular domain, we chose to focus initially on the ActRIIA type II BMP receptor. Analysis of the crystal structure of BMP7 bound to the ActRIIA ECD predicts that amino acid *Lys^76^* (PDB: 1LX5 for numbering) in ActRIIA is likely to interact with *Arg^48^* in BMP7, whereas the equivalent residue in BMP6, *Gln^48^*, would be unlikely to interact at this site (see below; Song et al., 2010; Perron and Dodd, 2012). We therefore next explored the role of *Lys^76^* in ActRIIA and the equivalent residue in ActRIIB using ActRIIA V3, an ActRIIA receptor variant in which three different codons for the amino acid Valine were altered within the *sh-AIIA* targeted sequence, producing a *sh-AIIA*-resistant ActRIIA receptor subunit (Fig. 4A and Fig. S3). In ActRIIA, *Lys^76^* was swapped for alanine (A), to provide a generic amino acid substitution (ActRIIA K76A), and for glutamic acid (E), to substitute the equivalent amino acid in ActRIIB (ActRIIA K76E) (Fig. 4A and Fig. S3). To determine first whether the ActRIIA V3 K76A and ActRIIA K76E variants are functional receptor subunits, BMP-responsive C2C12 mouse myoblast cells were transfected with empty vector (pcDNA3.1), ActRIIA V3, ActRIIA V3 K76A and ActRIIA V3 K76E cDNA expression plasmids. Western blot analysis of transfected whole-cell lysates demonstrates that ActRIIA V3 K76A and ActRIIA V3 K76E are expressed at levels equivalent to levels of ActRIIA V3 (Fig. 4B). The ActRIIA V3 K76A and ActRIIA V3 K76E plasmids were also evaluated for their ability to substitute for ActRIIA in receptor complexes and mediate activation of pSmad1/5/8 by treating transfected C2C12 cells with 50 ng/ml BMP7 for 30 min. Comparable increases in the levels of pSmad1/5/8 were observed in cells expressing ActRIIA V3, ActRIIA V3 K76A and ActRIIA V3 K76E (Fig. 4C), indicating that the K76 variants form functional receptor complexes. Moreover, the results confirm that type I BMP receptor-dependent signaling through Smad1/5/8 is unaffected by sequence changes at *Lys^76^* in ActRIIA.

**Fig. 5.**
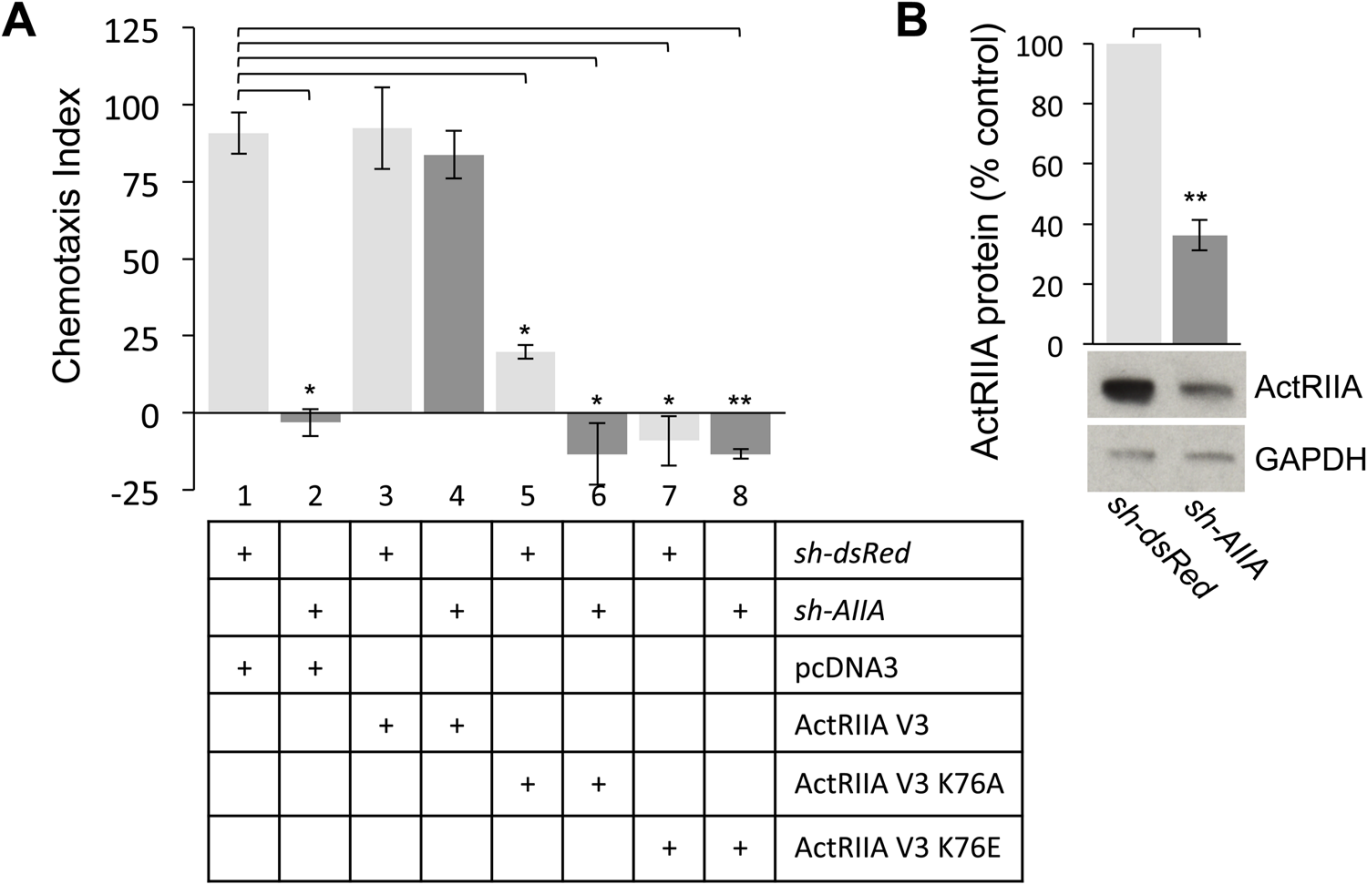
**ActRIIA V3 K76 receptor variants cannot rescue *sh-AIIA*-mediated inhibition of BMP7-stimulated chemotaxis.** (A) Chemotaxis in response to 50 ng/mL BMP7 (CI={[(no. BMP7 treated cells in filter pores)–(no. control cells in filter pores)]/(no. control cells in filter pores)}×100; mean±s.e.m.). Chemotaxis indices for dsRed^Δ^WEHI cells co-expressing pcDNA3 (lane 1, CI=90.9±6.8), ActRIIA V3 (lane 3, CI=92.3±13.2), ActRIIA V3 K76A (lane 5, CI=19.8±2.15) and ActRIIA V3 K76E (lane 7, CI=−4.5±9.5) (*n*=2). Chemotaxis indices for AIIA^Δ^WEHI cells co-expressing pcDNA3 (lane 2, CI=−3.1±4.4), ActRIIA V3 (lane 4, CI=83.8±7.7), ActRIIA V3 K76A (lane 6, CI=−13.3±10) and ActRIIA V3 K75E (lane 8, CI=−13.4±1.6). BMP7-evoked chemotaxis in dsRed^Δ^WEHI cells co-expressing pcDNA3 differs significantly from chemotaxis in dsRed^Δ^WEHI cells co-expressing ActRIIA V3 K76A and ActRIIA V3 K76E and in AIIA^Δ^WEHI cells co-expressing pcDNA3 and ActRIIA V3 K76A (*P<0.02, *n*=2) (Student's two-tailed *t*-test). The difference in BMP7-evoked chemotaxis between dsRed^Δ^WEHI cells co-expressing pcDNA3 and AIIA^Δ^WEHI cells co-expressing ActRIIA V3 K76E is also significant (**P<0.005, *n*=2) (Student's two-tailed *t*-test). (B) Western blots of dsRed^Δ^WEHI or AIIA^Δ^WEHI cell lysates co-expressing pcDNA3 were probed for ActRIIA expression. Detection of GAPDH provided a loading control. Electroporation of *sh-AIIA* in WEHI 274.1 cells decreases ActRIIA protein expression to 36%±5.1% of the expression levels in dsRed^Δ^WEHI lysates (mean±s.e.m.; *n*=4; **P<0.005, Student's two-tailed *t-*test).

We next examined the ability of the ActRIIA V3 K76 variants to participate in BMP7-dependent chemotropic activity using WEHI 274.1 monocytic cells in chemotaxis rescue assays. First, we confirmed knockdown of ActRIIA expression by *sh-AIIA* in FACS-sorted, GFP-enriched populations of WEHI 274.1 cells. Western blots of whole-cell lysates probed with an anti-ActRIIA antibody showed a 64% reduction in ActRIIA protein expression in AIIA^Δ^WEHI cells compared with dsRed^Δ^WEHI cells (Fig. 5B). Sorted WEHI cells expressing either *sh-dsRed* or *sh-AIIA* in combination with pcDNA3.1, ActRIIA V3 or the ActRIIA K76 variants were used in chemotaxis assays to determine the Chemotaxis Index (CI) as described in the Materials and Methods. As previously shown, chemotaxis stimulated by BMP7 is inhibited in AIIA^Δ^WEHI cells (Fig. 5A, lane 2, CI=−3) and this inhibition is rescued by co-expression of the shRNA-resistant expression plasmid, ActRIIA V3 (Fig. 5A, lane 4, CI=84; see also Perron and Dodd, 2009). Co-expression of either pcDNA3 or ActRIIA V3 had no effect on chemotaxis of dsRed^Δ^WEHI cells stimulated by BMP7 (Fig. 5A, lanes 1 and 3, CI=91 and 92, respectively). In contrast, dsRed^Δ^WEHI cells co-expressing ActRIIA V3 K76A or ActRIIA V3 K76E, a condition in which endogenous ActRIIA continues to be expressed, significantly inhibited BMP7-evoked chemotaxis (Fig. 5A, lanes 5 and 7, CI=20 and −4.5, respectively). This inhibition was even more dramatic in AIIA^Δ^WEHI cells co-expressing the K76 variants (Fig. 5A, lanes 6 and 8, CI=−13 for both), a condition in which endogenous ActRIIA is significantly reduced.

A reciprocal receptor variant, ActRIIB E75K, was generated by swapping *Glu^75^* in ActRIIB with the equivalent amino acid residue (K) in ActRIIA (see Fig. 4A and Fig. S3). As we have previously shown, expression of ActRIIB in AIIA^Δ^WEHI cells fails to rescue BMP7-evoked chemotaxis (Fig. S4, lane 2, CI=8.7) and has no effect on the response of dsRed^Δ^WEHI cells to BMP7 (Fig. S4, lane 1, CI=42). In contrast, co-expression of ActRIIB E75K in dsRed^Δ^WEHI cells significantly inhibited BMP7-evoked chemotaxis even in the presence of endogenous ActRIIA (Fig. S4, lane 3, CI=20). In AIIA^Δ^WEHI cells, the expression of ActRIIB E75K further suppresses the response to BMP7 (Fig. S4, lane 4, CI=−16) indicating that the presence of lysine at this site is not sufficient to allow chemotaxis by ActRIIB.

### Alteration of *Lys^76^* in ActRIIA blocks PI3K-dependent but not Smad-dependent downstream signaling

**Fig. 6.**
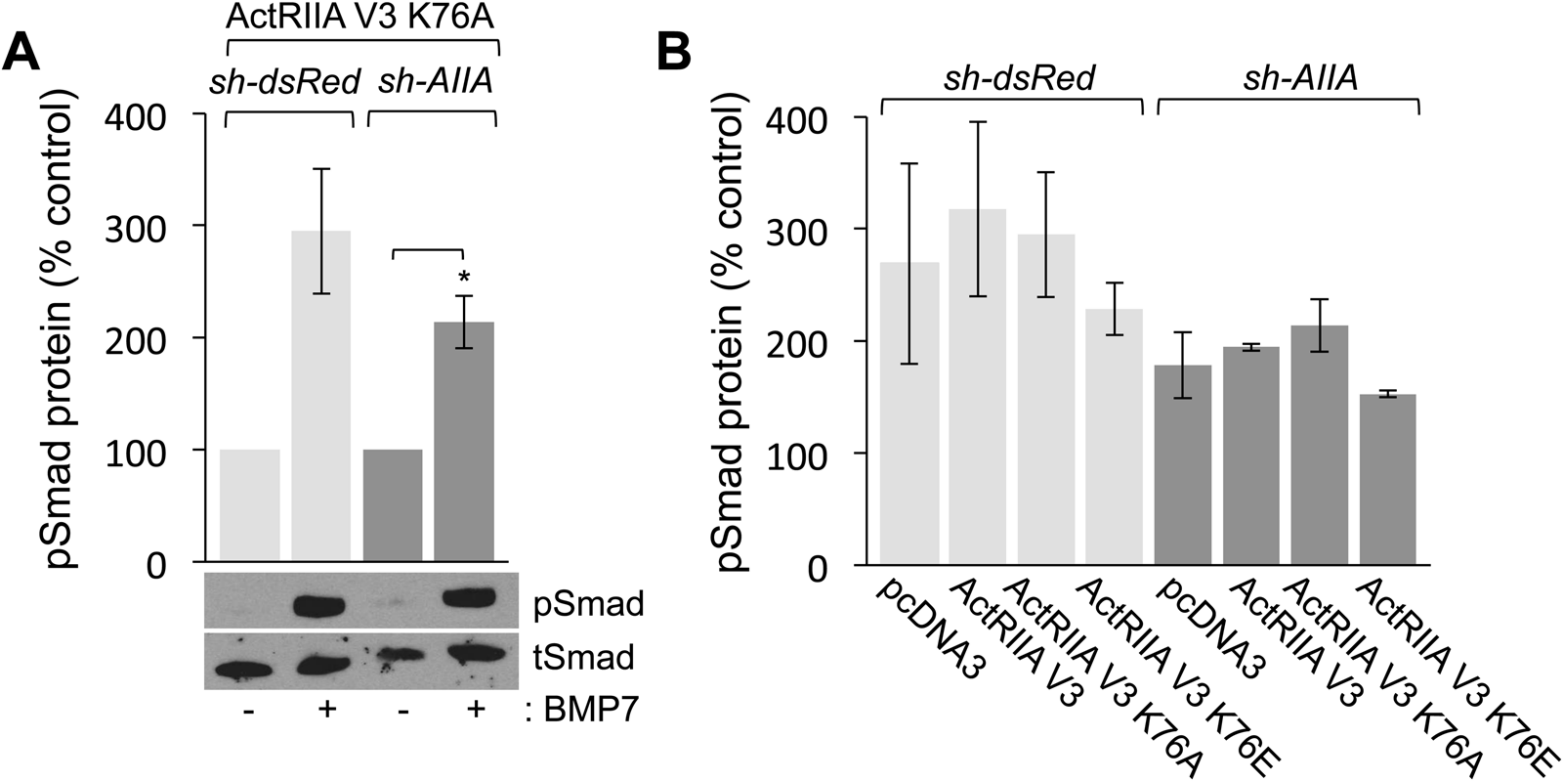
**Expression of ActRIIA V3 K76 variant receptors does not inhibit BMP7-evoked Smad1/5/8 phosphorylation.** (A) Western blots of dsRed^Δ^WEHI or AIIA^Δ^WEHI lysates co-expressing ActRIIA V3 K76A incubated with or without 50 ng/ml BMP7 for 30 min and probed for pSmad1/5/8. Detection of tSmad provided a loading control. BMP7 stimulated robust increases (mean±s.e.m.) in the levels of pSmad1/5/8 in dsRed^Δ^WEHI (195% over control, *n*=2) or AIIA^Δ^WEHI (113% over control, *n*=2; *P<0.05, Student's two-tailed *t*-test) cells co-expressing ActRIIA V3 K76A. (B) Quantification of western blots (mean±s.e.m.) of dsRed^Δ^WEHI or AIIA^Δ^WEHI lysates co-expressing pcDNA3, ActRIIA V3, ActRIIA V3 K76A and ActRIIA V3 K76E incubated in the presence of 50 ng/ml BMP7 for 30 min and probed for pSmad1/5/8. Detection of tSmad provided a loading control. Levels of pSmad1/5/8 were normalized to pSmad1/5/8 levels in unstimulated cells. All conditions demonstrated increases in response to BMP7 stimulation compared with control cells. The data for the unstimulated controls are not shown. There was no significant difference in the level of pSmad1/5/8 in dsRed^Δ^WEHI lysates compared with pSmad1/5/8 levels in AIIA^Δ^WEHI cells for any of the transfection conditions.

The failure of ActRIIA V3 K76A to rescue BMP7-evoked chemotaxis in AIIA^Δ^WEHI cells implies that interaction with *Lys^76^* in ActRIIA is required for selective activation of downstream signaling leading to chemotropic responses. To determine the effect of ActRIIA V3 K76A variant expression on BMP7-evoked downstream signaling, we examined pSmad1/5/8 and pAkt levels in the same cells used in the chemotaxis assays shown in Fig. 5. Western blot analysis of dsRed^Δ^WEHI or AIIA^Δ^WEHI cells co-expressing the ActRIIA V3 K76A variant shows robust increases in pSmad1/5/8 levels in the presence of BMP7 (Fig. 6A, 195% and 113% over control, untreated cultures, respectively). For each condition examined, the loss of ActRIIA expression and/or expression of the *Lys^76^* variant receptors did not interfere with BMP7-stimulated Smad1/5/8 phosphorylation, when compared with cells expressing pcDNA3 or ActRIIA V3 (Fig. 6B). This suggests that the loss of chemotropic activity observed in monocyte chemotaxis assays is independent of type I BMP receptor activity.

**Fig. 7.**
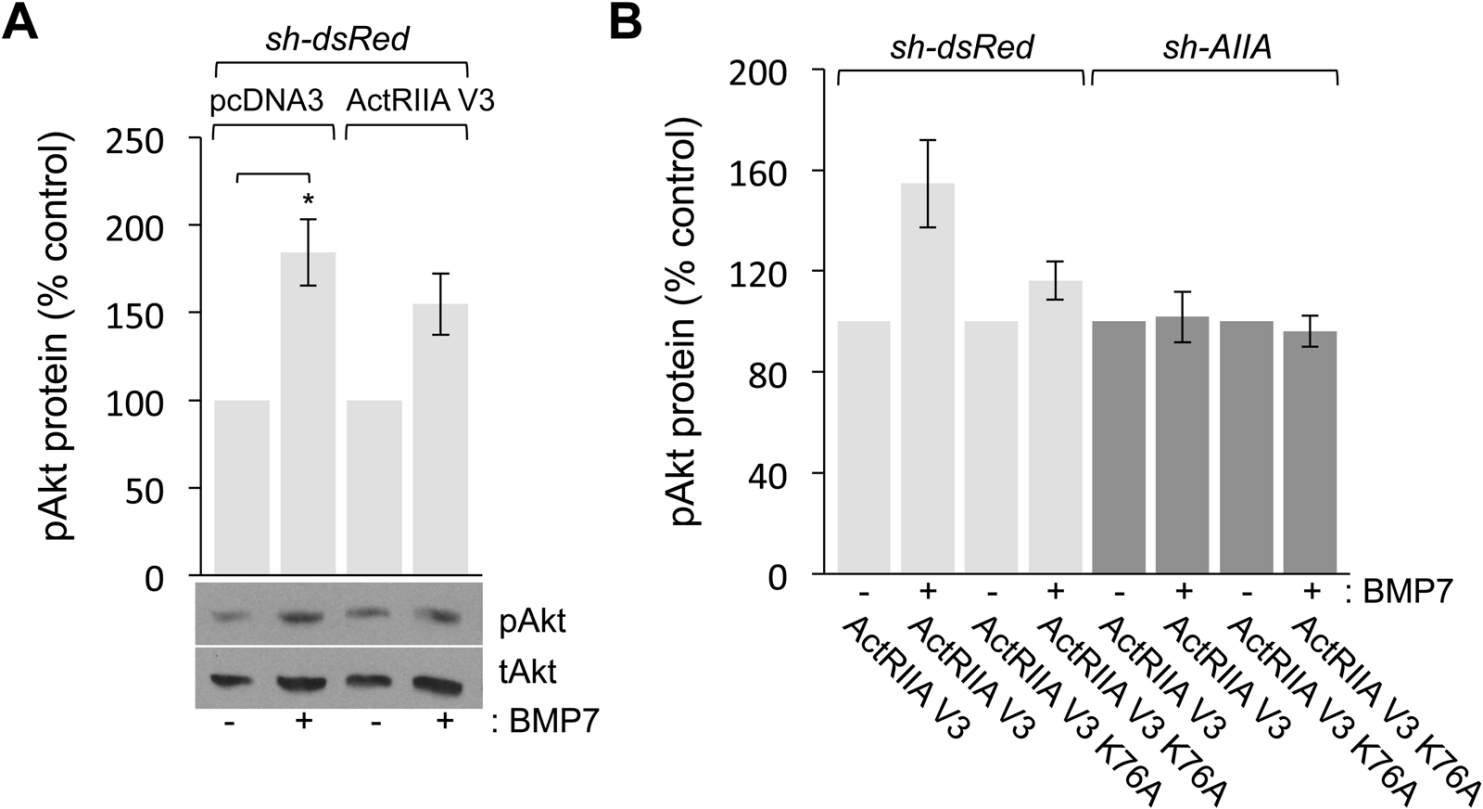
**BMP7-evoked Akt phosphorylation is inhibited by loss of ActRIIA expression or expression of ActRIIA V3 K76A.** (A,B) Quantification (mean±s.e.m.; *n*=2) of western blots of dsRed^Δ^WEHI and AIIA^Δ^WEHI lysates co-expressing the indicated cDNA expression constructs incubated with or without 50 ng/ml BMP7 for 30 min and probed for pAkt. Detection of tAkt provided a loading control. pAkt levels for each transfection condition were normalized to pAkt levels in the respective unstimulated cells. (A) BMP7 stimulated increases in the levels of pAkt in dsRed^Δ^WEHI cells co-expressing pcDNA3 (84%±19% over control; *P<0.05, Student's two-tailed *t*-test) or ActRIIA V3 (55%±17% over control). (B) BMP7 stimulated increases in the levels of pAkt only in dsRed^Δ^WEHI co-expressing ActRIIA V3 (55%±17% over control, lane 2). Increases in pAkt over control levels were not observed in any other condition in response to BMP7 including in dsRed^Δ^WEHI co-expressing ActRIIA V3 K76A (16%±7.5% over control, lane 4), AIIA^Δ^WEHI co-expressing ActRIIA V3 (1.8%±4.7% over control, lane 6) or AIIA^Δ^WEHI co-expressing ActRIIA V3 K76A (−3.8%±6.2% over control, lane 8).

Examination of BMP7-stimulated pAkt levels revealed dramatic alteration in PI3K-dependent signaling in the presence of the ActRIIA K76A variant. In control, dsRed^Δ^WEHI cells co-expressing pcDNA3 or ActRIIA V3, stimulation with BMP7 increased pAkt levels to 84% and 55% over control, respectively (Fig. 7A). In contrast, BMP7-stimulated Akt phosphorylation was significantly altered by the loss of endogenous ActRIIA expression or the expression of ActRIIA K76A. In Fig. 7B, the data are reported with respect to the individual, unstimulated cells for each condition. Stimulation of pAkt in dsRed^Δ^WEHI cells expressing ActRIIA V3 from Fig. 7A is shown for comparison and demonstrates BMP7-stimulated increases in pAkt (55% over control; Fig. 7B, lanes 1 and 2). In contrast, BMP7 was unable to stimulate an increase in pAkt in dsRed^Δ^WEHI cells co-expressing ActRIIA V3 K76A (16% over control; Fig. 7B, lanes 3 and 4). BMP7 also failed to stimulate pAkt over control levels in AIIA^Δ^WEHI cells expressing ActRIIA V3 (1.8% over control; Fig. 7B, lanes 5 and 6) or ActRIIA V3 K76A (3.8% below control; Fig. 7B, lanes 7 and 8). Although BMP7 failed to stimulate increases in pAkt in cells expressing either *sh-AIIA* or ActRIIA V3 K76A, we, nevertheless observed higher levels of pAkt in these samples.

To demonstrate this effect more clearly, the levels of Akt phosphorylation were recalculated with respect to pAkt levels in unstimulated WEHI cells co-expressing *sh-dsRed* and ActRIIA V3. These cells behave like wild-type cells in monocyte chemotaxis assays (compare lanes 1 and 3 in Fig. 5A), Smad phosphorylation (compare lanes 1 and 2 in Fig. 6B) and Akt phosphorylation (compare lanes 1 and 2 in Fig. 7A). Comparison of the relative pAkt levels in dsRed^Δ^WEHI and AIIA^Δ^WEHI cells co-expressing ActRIIA V3 showed that loss of endogenous ActRIIA expression resulted in increased levels of Akt phosphorylation, even in the absence of BMP7 stimulation (Fig. S5, compare lanes 1 and 3). The increase of basal pAkt levels was observed in all conditions in which *sh-AIIA* was expressed (Fig. S5, lanes 3, 4, 7 and 8). Expression of ActRIIA V3 K76A in dsRed^Δ^WEHI also resulted in increased pAkt levels in the absence of BMP7 (Fig. S5, compare lanes 1 and 5). Moreover, this effect on basal pAkt levels is increased even further by co-expression of ActRIIA V3 K76A in AIIA^Δ^WEHI cells (Fig. S5, compare lanes 1 and 7), suggesting an important link between *Lys^76^* in ActRIIA and the regulation of PI3K activity.

These results reveal important structural requirements in the type II BMP receptor, ActRIIA, which impact chemotropic, PI3K-dependent signaling stimulated by BMPs without affecting type I BMP receptor Smad-dependent signaling. Disruption of the BMP7:ActRIIA binding interface, significantly and specifically affects BMP7-evoked chemotropic responses. Taken together, our findings support a model in which only a subset of BMPs are able to bind to ActRIIA in a manner that then permits engagement of the PI3K-dependent, chemotropic signaling pathway, resulting in monocyte chemotaxis or growth cone collapse.

## DISCUSSION

**Fig. 8.**
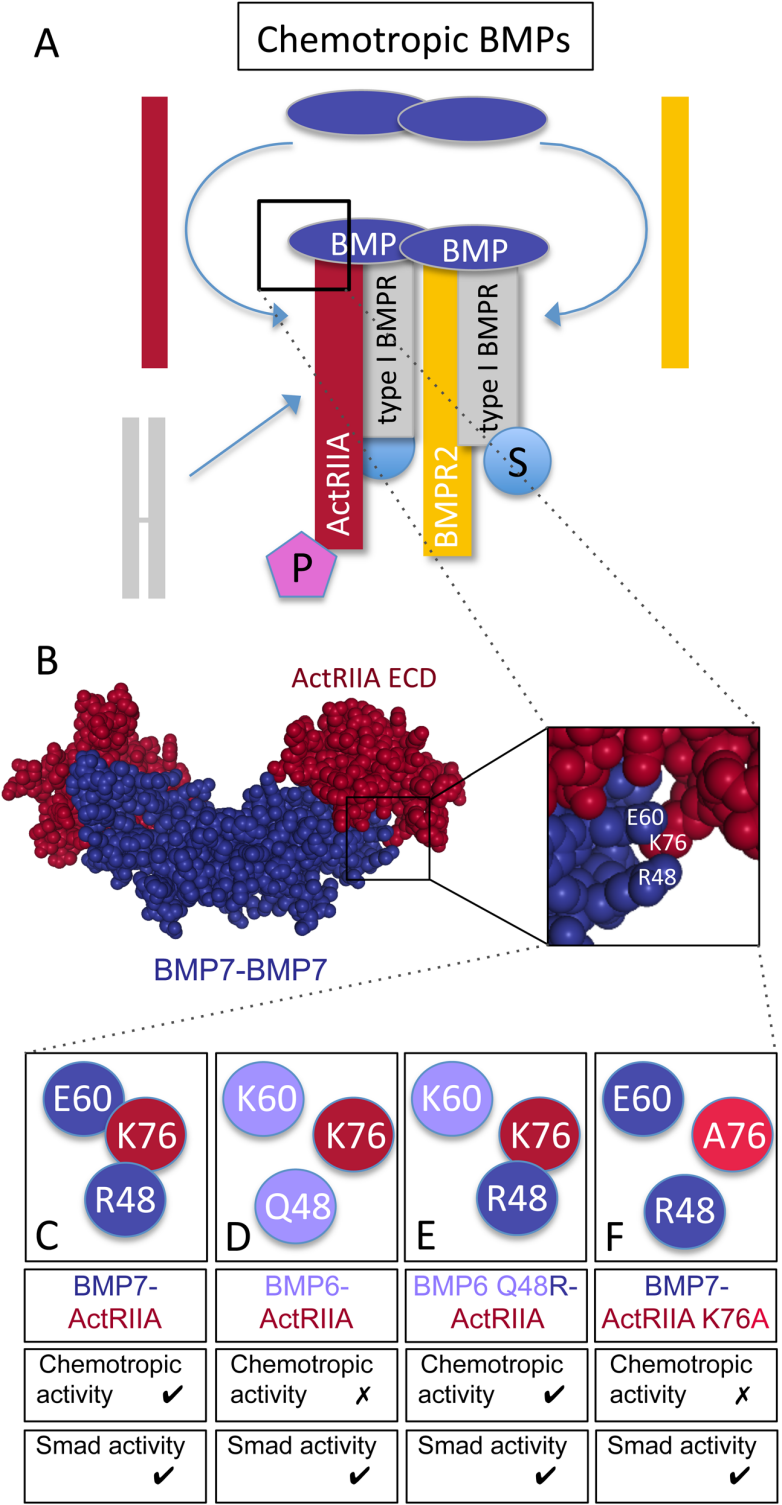
**Signaling and receptor binding interactions of chemotropic BMPs.** (A) Model of chemotropic BMP signaling indicating the potential asymmetric recruitment of ActRIIA (red) and BMPR2 (yellow) into the tetrameric receptor complex. A type I BMP receptor pair is represented as a preformed complex (PFC, gray subunits), though it is unclear whether BMP7 selectively engages PFCs containing ActRIIA and BMPR2 or recruits individual receptors into a complex with type I BMP receptor subunits. BMP7 stimulates Smad- (S) and PI3K-dependent (P) downstream signaling linked to type I and type II BMP receptors, respectively. (B) Spacefill representation of the crystal structure of a BMP7 dimer (blue) bound to the ActRIIA ECD (red) generated from Protein Data Bank ID, 1LX5 (Greenwald et al., 2003) using NGL Viewer (Rose et al., 2018). Enlarged area indicates amino acid positions for BMP7 R48 and E60 as well as ActRIIA K76. (C–F) Representations of wild-type and mutated amino acids in BMP-ActRIIA interactions and the associated chemotropic and Smad activity. (C) BMP7 R48 is predicted to associate with ActRIIA K76 and BMP7-evoked chemotropic activity requires interaction with ActRIIA. (D) BMP6 Q48 is not predicted to associate with ActRIIA K76 and BMP6 does not stimulate chemotropic activity. (E) BMP6 Q48R demonstrates potent chemotropic activity and would potentially interact with ActRIIA K76. (F) Mutation of K76 in ActRIIA to alanine (A76) is predicted to disrupt the interaction with BMP7 R48. The presence of ActRIIA K76A blocks BMP7-evoked chemotropic activity. Mutations at this site do not have any effect on BMP-stimulated Smad phosphorylation.

Classically, functional BMP receptor complexes have been shown to comprise of a pair of type I and a pair of type II BMP receptors, bound by a dimeric BMP (Fig. 8A; Miyazono et al., 2010). Once the ligand/receptor complex is assembled, the type II BMP receptors phosphorylate and activate type I BMP receptors resulting in the disassociation of activated Smad1/5/8 transcription factors. This mechanism assumes a perfunctory role for the type II BMP receptors. Indeed, type II BMP receptors are reported to harbor a constitutively active kinase domain and thus have been thought to serve only to associate with and activate type I BMP receptors (Shi and Massagué, 2003). However, distinct roles for type II BMP receptor subunits that are independent of the canonical Smad pathway have been demonstrated (Foletta et al., 2003; Lee-Hoeflich et al., 2004; Perron and Dodd, 2009, 2011). Our previous studies have shown that in a monocytic cell line, BMP7-evoked monocyte chemotaxis is dependent on two specific type II BMP receptors, ActRIIA and BMPR2 (Perron and Dodd, 2009). We now have explored structural and functional requirements of type II BMP receptors for BMP7-mediated growth cone collapse in DSC neurons. Our results show that BMP7-evoked growth cone collapse requires ActRIIA and BMPR2, but not ActRIIB; subunit requirements consistent with our findings in BMP7-evoked monocytic chemotaxis. The potential for structural interactions at the BMP7:ActRIIA interface led us to explore and demonstrate that chemotropic activity stimulated by BMP7 through ActRIIA requires the presence of a specific amino acid residue in the ActRIIA receptor subunit. Alteration of *Lys^76^* in ActRIIA to *Ala^76^* (Fig. 8F) or *Glu^76^* prevents BMP7-evoked monocyte chemotaxis. Consistent with the idea that ActRIIA is required selectively for chemotropic activity but not transcriptional responses, amino acid substitutions at *Lys^76^* in ActRIIA had no effect on BMP7-stimulated increases in pSmad1/5/8 levels. In contrast, the loss of ActRIIA expression or expression of the ActRIIA K76A variants eliminated the ability of BMP7 to regulate Akt phosphorylation. These results demonstrate a common requirement for specific type II BMP receptor subunits, ActRIIA and BMPR2, in BMP7-mediated chemotropic activity in widely different cell types, monocytes and neurons. This work also reveals structural determinants in ActRIIA specifically related to the chemotropic actions of BMP7 and provides further evidence for an active role for type II BMP receptor subunits, distinct from the role of type I BMP receptors, in mediating intracellular signaling in response to BMPs, specifically leading to cytoskeletal reorganization initiated by chemotropic BMPs.

### Improved transfection efficiency with whole-embryo electroporation

Standard transfection techniques based on lipofection methods are particularly inefficient in cultures of dissociated primary neurons. We developed a method to introduce shRNAs into DSC neurons by whole-embryo electroporation. This method has a number of advantages over standard lipofection techniques. As shown in Fig. 1, whole-embryo electroporation results in significantly higher transfection efficiencies than lipofection. Moreover, liposome-mediated transfection is often toxic to cells, especially over extended incubation times, and requires a number of medium changes, adding to cellular stress (Dodds et al., 1998; Gharaati-Far et al., 2018). These issues are eliminated when carrying out electroporation techniques as no reagents other than the nucleic acid is required. Furthermore, transfection of shRNA vectors or cDNA expression plasmids can be targeted to specific regions of the developing embryo permitting introduction of the constructs into the target cells *in situ* before isolation, dissociation and seeding for culture. Our study demonstrates the success of whole-embryo electroporation as a tool to investigate molecular events in individual neurons, revealing the structural requirements for individual type II BMP receptor subunits in BMP-dependent chemotropic activity by RNAi in DSC neurons.

### ActRIIA K76 is an important specificity determinant for BMP7-evoked chemotropic activity

Two major sites of interaction with type I and type II BMP receptors have been identified in BMP dimers (known as the wrist epitope for type I receptors and the knuckle for type II) (Sebald et al., 2004; Yadin et al., 2016). At these sites, the interacting moieties are likely to have a variety of functions. Only a few amino acid substitutions at BMP ligand/receptor epitopes have a direct effect on binding (Kirsch et al., 2000; Weber et al., 2007). In contrast, single amino acid substitutions in either BMPs or BMP receptors can significantly change signaling outcomes without affecting binding (Kirsch et al., 2000; Nickel et al., 2005; Allendorph et al., 2007; Song et al., 2010; Perron and Dodd, 2012), suggesting a role for conferring signaling specificity. In the current experiments, substitution of *Lys^76^* with *Ala^76^* (Fig. 8F) did not inhibit expression of the ActRIIA receptor variant or the ability to respond to BMP7. The ActRIIA K76A substitution did, however, significantly affect the nature of the response of the receptor complex to BMP7, allowing Smad-dependent signals to be transduced while blocking BMP7-evoked increases in pAkt.

We have previously shown that *Arg^48^* in BMP7 is an important determinant of chemotropic activity stimulated by BMP7 (Fig. 8C). Analysis of the BMP7/ActRIIA ECD complex (PDB: 1LX5) predicts an interaction between *Arg^48^* in BMP7 and *Lys^76^* in ActRIIA (Fig. 8B,C; Song et al., 2010; Perron and Dodd, 2012). A number of studies have implicated *Lys^76^* in ActRIIA in high affinity binding of BMP7 to ActRIIA (Shah et al., 2001; Allendorph et al., 2007; Song et al., 2010). Other studies have reported an amino acid in BMP2 (*Ser^24^*), corresponding to *Arg^48^* in BMP7, is likely to interact with residues at the type I BMP receptor interface (Greenwald et al., 2003; Schreuder et al., 2005). However, Schreuder et al. (2005) proposed that *Arg^48^* in BMP7 is buried by both type I and type II BMP receptors in the hexameric complex suggesting that *Arg^48^* may have a role in coordinating both type I and type II BMP receptor subunit assembly into the chemotropic receptor complex. The non-chemotropic BMP, BMP6, harbors a glutamine at position 48 (*Gln^48^*) and is not expected to interact with *Lys^76^* in ActRIIA (Fig. 8D). This might hamper recruitment of ActRIIA into the chemotropic receptor complex or prevent the ActRIIA subunit from assuming the conformation necessary for relaying chemotropic signals through PI3K-dependent pathways. Indeed, replacing *Gln^48^* in BMP6 with *Arg^48^* from the BMP7 sequence converts BMP6 into a chemotropic BMP with growth cone collapsing activity equivalent to that of BMP7 (Fig. 8E; Perron and Dodd, 2012). These findings support the idea that the interaction between *Arg^48^* in BMP7 and *Lys^76^* in ActRIIA is a critical determinant of BMP7-evoked chemotropic activity; though the mechanism underlying this function is not clear. Moreover, the surprising upregulation of pAkt in cells lacking ActRIIA or expressing ActRIIA V3 K76A reflects unregulated PI3K activity and suggests that the ActRIIA subunit plays a critical role in regulating PI3K-dependent signaling.

### A common receptor complex for chemotropic BMPs

Of the more than 20 BMP family members identified, only a small subset, BMP2, BMP4, BMP7 and BMP9, have been demonstrated to promote rapid, chemotropic responses that are distinct from the long-term effects following stimulation of gene transcription pathways (Augsburger et al., 1999; Wen et al., 2007; Gamell et al., 2008; Perron and Dodd, 2009, 2012). We have shown that both BMP7-evoked chemotaxis in monocytes and growth cone collapse in DSC neurons require the activity of two distinct type II BMP receptor subunits. A requirement for the same type II BMP receptor subunits mediating the activity of BMP9 in endothelial cells has also been reported (Upton et al., 2009; Kim et al., 2012). These data support the idea of selective, asymmetric recruitment of the type II BMP receptor subunits, ActRIIA and BMPR2, into a distinct ligand/receptor complex (Fig. 8A), coupling BMP7, and potentially other chemotropic BMPs, to cytoskeletal regulatory machinery. Type I BMP receptor activity, however, is not required for BMP7-evoked chemotropic activities (current study and Perron and Dodd, 2009, 2011).

How the subunits of the BMP receptor complex are assembled has been and remains a major question (Nohe et al., 2002; Greenwald et al., 2003; Heinecke et al., 2009; Ehrlich et al., 2011). There are two demonstrated modes of BMP receptor assembly: 1) ligand binding to preformed complexes of receptor pairs (PFCs) or 2) BMP-induced signaling complexes (BISCs) (Fig. 8A). Moreover, the mode appears to dictate the downstream signaling response, with Smad-dependent signaling occurring after BMP engagement with PFCs and non-Smad-dependent pathways stimulated through BISCs (Nohe et al., 2002; Sieber et al., 2006; Nickel et al., 2009; Marom et al., 2011).

All possible homomeric and heteromeric combinations of BMPR2, ALK3 and ALK6 PFCs have been detected in unstimulated cells expressing epitope-tagged BMP receptors and these combinations are altered in response to BMP stimulation (Gilboa et al., 2000; Ehrlich et al., 2011; Marom et al., 2011). It is unclear whether ActRIIA and BMPR2 can also form hetero-oligomeric PFCs. Such a subunit combination would be an attractive target for recruitment by BMP7 into a chemotropic receptor complex. Heterodimeric BMP ligands may be ideally suited to recruit different BMP receptor subunits into a signaling complex. Indeed, BMP2:BMP7 heterodimers display greater potency than homodimers of either BMP2 or BMP7 in both *in vivo* and *in vitro* assays of bone and joint repair (Sun et al., 2012; Li et al., 2017). Moreover, BMP2:BMP7 heterodimers have been shown asymmetrically to recruit distinct type I BMP receptors into a complex that mediates patterning of the dorsoventral axis in *Drosophila* embryos (Little and Mullins, 2009). Significantly, a BMP7:GDF7 heterodimer repels DSC axons in *in vitro* reorientation assays and appears to direct the initial trajectory of developing DSC neurons *in vivo* (Butler and Dodd, 2003). These findings support the possibility that the BMP7:GDF7 heterodimer asymmetrically recruits ActRIIA and BMPR2 into a signaling complex that guides the early projections of DSC neurons.

In summary, we have revealed a mechanism for transduction of BMP7-evoked chemotropic activity that requires specific engagement of the type II BMP receptors ActRIIA and BMPR2. Our current findings identify *Lys^76^* in ActRIIA as an important determinant in the assembly of, and/or chemotropic signaling by, receptor complexes, leading to signaling to the cytoskeleton through PI3K-dependent pathways and leaving Smad-dependent signaling unaffected. This core mechanism of BMP-induced receptor recruitment and signaling by the chemotropic BMP, BMP7, underlies both the chemoattraction of monocytic cells and the chemorepulsion of growth cones of spinal sensory neurons and implies that cell context-specific pathway components or co-receptors may be involved to regulate the direction of the response.

## MATERIALS AND METHODS

### Antibodies and reagents

Recombinant BMP7 was purchased from R&D Systems (Minneapolis, MN, USA). Antibodies: mouse anti-ERM (Ezrin/Radixin/Moesin) IgM (13H9; Goslin et al., 1989); rabbit anti-GFP IgG (Invitrogen); rabbit anti-ActRII IgG (H65) and HRP-conjugated secondary antibodies (Santa Cruz Biotechnology); rabbit anti-phospho-Smad1/5/8 IgG (pSmad), rabbit anti-Smad1 IgG (tSmad), rabbit anti-phopho-Akt IgG (pAkt) and rabbit anti-Akt IgG (tAkt) (Cell Signaling Technology); rabbit anti-GAPDH IgG (Abcam); Cy3- and Cy2-conjugated secondary antibodies (Jackson ImmunoResearch Laboratories). The dilutions for each antibody used in the study are listed in Table S1. Cell culture reagents: Ham's F12 medium, DMEM high-glucose medium, 100× Penicillin/Streptomycin/Glutamine (PSG) (Invitrogen), FBS (Gemini BioProducts, West Sacramento, CA, USA). The expression construct for flag-tagged, mouse ActRIIA.pcDNA3 was generously provided by Dr K. Miyazono (The JFCR Cancer Institute, Japan). Site-directed mutagenesis of ActRIIA V3 at *Lys^76^* and of ActRIIB at *Glu^75^* was carried out (see Fig. 4) using the QuikChange Site-Directed Mutagenesis Kit (Agilent Technologies, Santa Clara, CA, USA).

### shRNA vectors

For RNAi-mediated downregulation of type II BMP receptor expression, shRNA vectors (*sh-dsRed*, *sh-AIIA*, *sh-AIIB* and *sh-BRII*) that target dsRed (negative control), ActRIIA, ActRIIB and BMPR2, respectively, were constructed and used as previously described (Perron and Dodd, 2009). BMP-stimulated transcriptional signaling was inhibited using an shRNA vector targeting Smad4 (*sh-Smad4*). Each of the constructs were previously validated for target specificity at the RNA and protein level by qPCR and western blot analysis, respectively. Briefly, shRNA oligonucleotides were cloned into the LentiLox3.7 lentiviral vector (pLL3.7, generously provided by Dr L. Van Parijs, MIT, USA; Rubinson et al., 2003) and were expressed under the control of the U6 promoter. In pLL3.7, concurrent EGFP expression under the control of the CMV promoter allows for identification of shRNA-expressing cells (see Figs 1 and 2).

### Embryo electroporation and dissociated culture

Concentrated shRNA vector DNA (1–2 µL of >10 mg/ml), along with Fast Green dye to visualize the DNA, was injected into the lumen of embryonic day 13 (E13) rat embryo spinal cords. Three pulses of 35 V in 50 ms intervals were applied using 3 mm L-shaped gold-tip Genetrodes (BTX, Holliston, MA, USA) placed on either side of the spinal cord, spanning the cervical-to-lumbar region of the embryo. Following a recovery period on ice for 1 h, the embryos were further dissected to isolate the electroporated side of the dorsal spinal cord. The electroporated dorsal spinal tissue was subsequently dissociated into a single cell suspension and plated onto poly-D-lysine/laminin-coated culture dishes (western blot analysis) or 12 mm glass coverslips (immunolabeling) as previously described (Augsburger et al., 1999; Perron and Dodd, 2011).

Electroporated DSC neurons were sorted as described below to generate whole-cell lysates enriched for GFP^+^, shRNA^+^ cells. For lipofection of E13 dissociated DSC cultures, neurons were transfected with shRNA vectors using Lipofectamine LTX (Invitrogen).

### Cell lines and cell sorting

The BMP-responsive C2C12 mouse myoblast cell line (ATCC, Rockville, MD, USA) was maintained in DMEM/10% FBS/PSG and transfected using the TransIT LT1 reagent (Mirus, Madison, WI, USA). The WEHI 274.1 mouse monocytic cell line (ATCC) was electroporated and sorted for GFP^+^ cells to enrich for shRNA-expressing cells, as previously described (Perron and Dodd, 2009). Briefly, WEHI 274.1 cells were electroporated and subjected to fluorescence-activated cell sorting (FACS) (Ultra Hypersort Flow Cytometer, Beckman Coulter, Atlanta, GA, USA). Sorted cells were subsequently used to generate whole-cell lysates or cultured for use in growth cone collapse, phosphorylation and chemotaxis assays.

### Growth cone collapse assays

Electroporated DSC cultures (shRNA^Δ^DSC) were serum-starved and stimulated with 50 ng/ml BMP7 for 30 min. The cultures were fixed and labeled with anti-GFP and anti-ERM antibodies as previously described (Perron and Dodd, 2011). The coverslips were mounted in Vectashield containing DAPI (Vector Labs, Burlingame, CA, USA) to label nuclei. The growth cone area of neurons with axons greater than 10 μm was measured using ImageJ 1.49v software. Growth cone area was measured from a total of at least six coverslips from at least three independent experiments with an average of 200 growth cones analyzed per condition. Growth cone collapsing activity is presented as the percentage of growth cone area decrease (GCAD) relative to control cultures, where GCAD=(100–{[(GC area in the presence of BMP7–control GC area)/control GC area]×100}).

### Phosphorylation assays

Serum-starved cultures (WEHI 274.1, C2C12 or DSC) were stimulated with and without 50 ng/ml BMP7 for 30 min, washed with ice-cold TBS and lysed in 1X Lysis Buffer (Cell Signaling Technology). Whole-cell lysates were used in western blot analysis in which membranes were probed with antibodies against pSmad1/5/8, tSmad, pAkt and tAkt.

### Chemotaxis assays

WEHI 274.1 monocytic cells were co-electroporated with *sh-dsRed* (dsRed^Δ^WEHI) or *sh-AIIA* (AIIA^Δ^WEHI) and either pcDNA3, ActRIIA V3, ActRIIA V3 K76A, ActRIIA V3 K76E, ActRIIB or ActRIIB E75K. The electroporated cells were sorted by FACS and used in phosphorylation assays (see above) or chemotaxis assays as previously described (Perron and Dodd, 2009). Chemotaxis counts were performed from duplicate filters; 40–20× fields per filter were counted. Results are presented as the chemotaxis index (CI)={[(no. treated cells in filter pores)–(no. control cells in filter pores)]/(no. control cells in filter pores)}×100.

### Western blot analysis

Whole-cell lysates were prepared using 1X Lysis Buffer (Cell Signaling Technology). Samples were separated by SDS-PAGE using either EZ-Run Gel Solution (Thermo Fisher Scientific) or TGX Fast Cast Acrylamide Kit (BioRad, Hercules, CA, USA) gels and transferred to nitrocellulose membranes (GE Healthcare Life Sciences). The membranes were blocked and probed as previously described (Perron and Dodd, 2009). The blots were developed using either the SuperSignal West Pico (Thermo Fisher Scientific) or Trident Pico Western HRP (GeneTex, Irvine, CA, USA) chemiluminescent substrates using Blue X-ray film (Phenix Research Products, Candler, NC, USA) or the Omega Lum G Imaging System (Gel Company, San Francisco, CA, USA). Densitometric analysis was performed using ImageJ 1.49v software.

### Imaging

Images of DSC cultures were taken with a Zeiss AxioCam HR digital camera (Carl Zeiss, Oberkochen, Germany) mounted on a Zeiss Axiovert 200M fluorescence microscope.

## Supplementary Material

Supplementary information
