# Supplementary material for: Chemotropic signaling by BMP7 requires selective interaction at a key residue in ActRIIA
Source: Biol Open. 2019 Jun 17;8(7):bio042283. doi: 10.1242/bio.042283 (PMC6679395; doi:10.1242/bio.042283)
Supplement: Supplementary information [file biolopen-8-042283-s1.pdf]

Table S1. Antibodies and dilutions used in this study.

Antibody	Source	Catalog #	Lot #	Dilution
Primary Antibodies				
pan-ERM (IgM)	Gift from F. Solomon (MIT)	Hybridoma supernatant (13H9)		1:200
GFP	Molecular Probes	A11122	1828014	1:1000
ACTR-II (H-65)	Santa Cruz Biotechnology	sc-25451	B2009	1:2000
GAPDH	Abcam	ab9485	808033	1:1000
Phospho-Smad1/5/9	Cell Signaling Technology	13820	3	1:4000
Smad1	Cell Signaling Technology	6944	5	1:4000
Phospho-Akt	Cell Signaling Technology	4060	23	1:8000
Akt (pan)	Cell Signaling Technology	4691	20	1:50,000
Secondary Antibodies				
HRP-conjugated mouse anti-rabbit IgG	Santa Cruz Biotechnology	sc-2357	I2215	1:10,000
Cy2-conjugated goat anti-mouse IgG	Jackson Immunoresearch Labs	115-225-146	133889	1:200
Cy3-conjugated goat anti-rabbit IgG	Jackson Immunoresearch Labs	111-165-144	134845	1:500
Cy3-conjugated goat anti-mouse IgM	Jackson Immunoresearch Labs	115-165-075	115625	1:500

Biology Open • Supplementary information

Figure S1. GFP expression is detected in transfected DSC neurons. Western blots of untransfected, whole cell DSC lysates and dsRed$^{\Delta}$DSC lysates were probed with anti-ActRIIA and anti-GFP antibodies demonstrating expression of GFP in transfected neurons only. Detection of GAPDH provided a loading control.

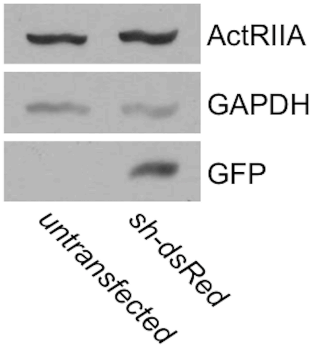

Figure S2. Knockdown of ActRIIA does not effect the expression of the remaining type II BMP receptors, ActRIIB and BMPR2. Western blots of dsRed$^{\Delta}$DSC and AIIA$^{\Delta}$DSC whole cell lysates were probed with anti-ActRIIA, anti-ActRIIB and anti-BMPR2 antibodies demonstrating selective loss of ActRIIA expression in the presence of *sh-AIIA*. Detection of GAPDH provided a loading control. This analysis also confirms that all three type II BMP receptors are expressed in DSC neurons.

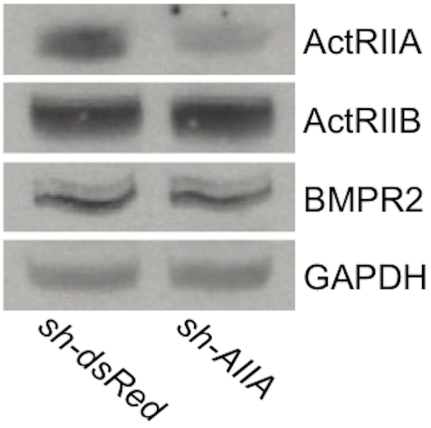

Biology Open • Supplementary information

Figure S3. Amino acid sequence alignment of mouse ActRIIA and mouse ActRIIB. The amino acid sequences for mouse ActRIIA (NCBI accession # NP_031422.3) and mouse ActRIIB (NCBI accession # NP_031423.1) were aligned using the NCBI BLAST Multiple Alignment tool. The *Lys* and *Glu* mutated in ActRIIA and ActRIIB, respectively, are highlighted in yellow and the *Val* residues mutated in ActRIIA V3 are highlighted in green. Note that the numbering for the full-length amino acid sequences shown here differs from numbering used throughout the paper, which is derived from the model of BMP7 bound to the ECD of ActRIIA (PDB ID: 1LX5; Greenwald et al., 2003).

```
ActRIIA 1   MGAAAKLAFA VFLISCSSGA ILGRSETQEC LFFNANWERD RTNQTGVEPC 50
ActRIIB 1   M-TAPWAALA LLWGSLCAGS GRGEAETREC IYYNANWELE RTNQSGLERC 49

ActRIIA 51  YGDKDKRRHC FATWKNISGS IEIVKQGCWL DDINCYDRTD CIEKKDSPEV 100
ActRIIB 50  EGEQDKRLHC YASWRNSSGT IELVKKGCWL DDFNCYDRQE CVATEENPQV 99

ActRIIA 101 YFCCCEGNMC NEKFSYFPEM EVTQPTSNPV TPKPPYYNIL LYSLVPLMLI 150
ActRIIB 100 YFCCCEGNFC NERFTHLPEP GGPEVTYEPP PTAPTLLTVL AYSLLPIGGL 149

ActRIIA 151 AGIVICAFWV YRHHKMAYPP VLV------- ---------- ------PTQD 177
ActRIIB 150 SLIVLLAFWM YRHRKPPYGH VDIHEVRQCQ RWAGRRDGCA DSFKPLPFQD 199

ActRIIA 178 PGPPPPSPLL GLKPLQLLEV KARGRFGCVW KAQLLNEYVA VKIFPIQDK  227
ActRIIB 200 PGPPPPSPLV GLKPLQLLEI KARGRFGCVW KAQLMNDFVA VKIFPLQDKQ 249

ActRIIA 228 SWQNEYEVYS LPGMKHENIL QFIGAEKRGT SVDVDLWLIT AFHEKGSLSD 277
ActRIIB 250 SWQSEREIFS TPGMKHENLL QFIAAEKRGS NLEVELWLIT AFHDKGSLTD 299

ActRIIA 278 FLKANVVSWN ELCHIAETMA RGLAYLHEDI PGLK-DGHKP AISHRDIKSK 326
ActRIIB 300 YLKGNIITWN ELCHVAETMS RGLSYLHEDV PWCRGEGHKP SIAHRDFKSK 349

ActRIIA 327 NVLLKNNLTA CIADFGLALK FEAGKSAGDT HGQVGTRRYM APEVLEGAIN 376
ActRIIB 350 NVLLKSDLTA VLADFGLAVR FEPGKPPGDT HGQVGTRRYM APEVLEGAIN 399

ActRIIA 377 FQRDAFLRID MYAMGLVLWE LASRCTAADG PVDEYMLPFE EEIGQHPSLE 426
ActRIIB 400 FQRDAFLRID MYAMGLVLWE LVSRCKAADG PVDEYMLPFE EEIGQHPSLE 449

ActRIIA 427 DMQEVVVHKK KRPVLRDYWQ KHAGMAMLCE TIEECWDHDA EARLSAGCVG 476
ActRIIB 450 ELQEVVVHKK MRPTIKDHWL KHPGLAQLCV TIEECWDHDA EARLSAGCVE 499

ActRIIA 477 ERITQMQRLT NIITTEDIVT VVTMVTNVDF PPKESSL               513
ActRIIB 500 ERVSLIRRSV NGTTSDCLVS LVTSVTNVDL LPKESSI               536
```

Figure S4. Mutation of ActRIIB *Glu*^{75} does not rescue BMP7-evoked chemotaxis. Chemotaxis in response to 50 ng/mL BMP7 (mean ± s.e.m.). Chemotaxis indices for dsRed$^{\Delta}$WEHI cells co-expressing ActRIIB (*lane 1*, CI = 41.7 ± 3.9) and ActRIIB E75K (*lane 3*, CI = 19.8 ± 2.2). Chemotaxis indices for AIIA$^{\Delta}$WEHI cells co-expressing ActRIIB (*lane 2*, CI = 8.7 ± 11) and ActRIIB E75K (*lane 4*, CI = -16 ± 1.0). In dsRed$^{\Delta}$WEHI cells co-expressing ActRIIB, BMP7-evoked chemotaxis differs significantly from chemotaxis in dsRed$^{\Delta}$WEHI cells co-expressing ActRIIB E75K (*P < 0.05, n = 2) and from chemotaxis in AIIA$^{\Delta}$WEHI cells co-expressing ActRIIB E75K (***P < 0.005, n = 2) (Student's two tailed t-test).

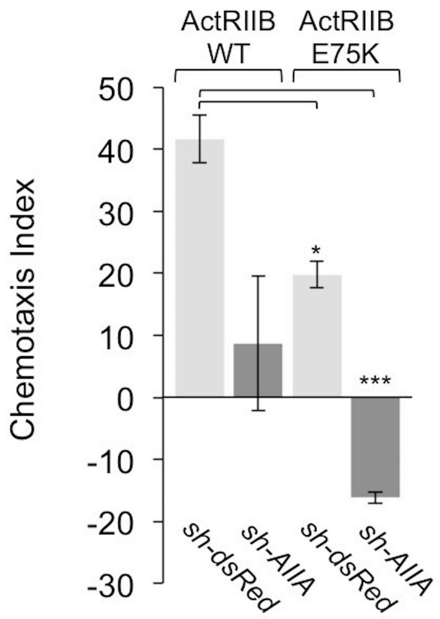

Biology Open • Supplementary information

Figure S5. Loss of ActRIIA and expression of ActRIIA K76A results in unregulated increases in pAkt levels. All samples are shown with respect to pAkt levels in unstimulated, dsRed$^{\Delta}$WEHI lysates expressing ActRIIA V3 (*lane 1*). Significant differences were observed in pAkt levels between unstimulated, dsRed$^{\Delta}$WEHI lysates co-expressing ActRIIA V3 and WEHI cells stimulated with BMP7 including AIIA$^{\Delta}$WEHI lysates co-expressing ActRIIA V3 (*lanes 1* and *4*, 82% over control), dsRed$^{\Delta}$WEHI lysates co-expressing ActRIIA V3 K76A (*lanes 1* and *6*, 87% over control) and AIIA$^{\Delta}$WEHI lysates co-expressing ActRIIA V3 K76A (*lanes 1* and *8*, 135% over control) (**P* < 0.05, Student's two tailed *t*-test). The difference in pAkt levels between dsRed$^{\Delta}$WEHI lysates co-expressing ActRIIA V3 (*lane 1*) and AIIA$^{\Delta}$WEHI lysates co-expressing ActRIIA V3 K76A (*lane 7*, 144% over control) in the absence of BMP7 is highly significant (****P* < 0.0001, Student's two tailed *t*-test). Increases in pAkt levels in the absence of BMP7 stimulation including AIIA$^{\Delta}$WEHI lysates co-expressing ActRIIA V3 (*lane 3*, 80% over control) and dsRed$^{\Delta}$WEHI lysates co-expressing ActRIIA V3 K76A (*lane 5*, 75% over control) were also observed.

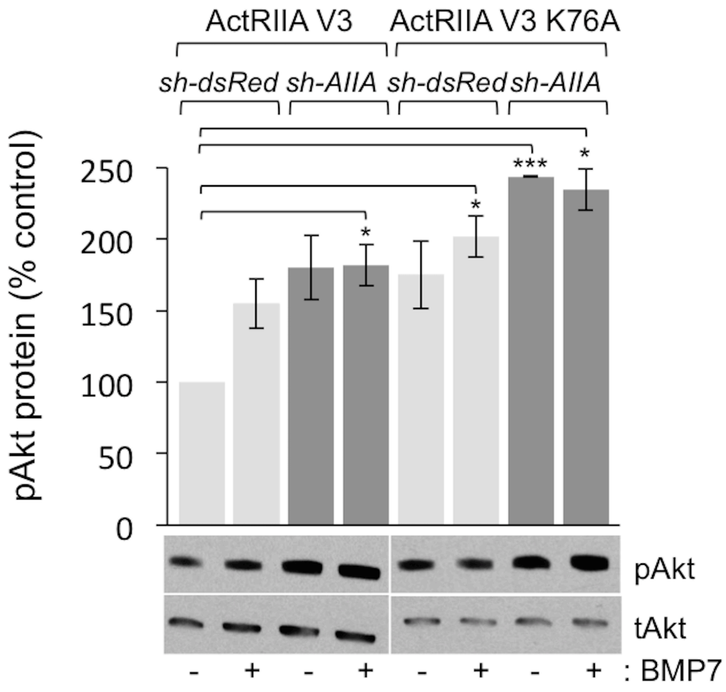

Biology Open • Supplementary information

List of Symbols and Abbreviations

ActRIIA	Activin A receptor type IIA
ActRIIB	Activin A receptor type IIB
ALK	Activin receptor-like kinase
BISC	BMP-induced signaling complex
BMP	Bone morphogenetic protein
BMPR2	BMP receptor type 2
CI	Chemotaxis index
DSC	Dorsal spinal cord
ECD	Extracellular domain
EGFP	Enhanced Green Fluorescent Protein
FACS	Fluorescence-activated cell sorting
GCAD	Growth cone area decrease
LIMK1	Lim domain kinase 1
PDB	Protein Data Bank
PI3K	Phosphoinositide 3-kinase
PFC	Preformed complex
RNAi	RNA interference
shRNA	short hairpin RNA
TBS	Tris-buffered saline
TGFβ	Transforming growth factor beta
